# Enhancing Catheter-Assisted C-Arm CT-Guided Ablation with PET/CT Fusion: A Pictorial Overview of Multimodal Synergy for Improving Local Tumor Control in Liver Metastasis

**DOI:** 10.3390/cancers18101584

**Published:** 2026-05-13

**Authors:** Laurens Hermie, Charlotte Harth, Kathia De Man, Alexander Decruyenaere, Celine Jacobs, Karen Geboes

**Affiliations:** 1 Department of Vascular and Interventional Radiology, Ghent University Hospital, 9000 Gent, Belgium; 2 Department of Nuclear Medicine, Ghent University Hospital, 9000 Gent, Belgium; 3 Department of Medical Oncology, Ghent University Hospital, 9000 Gent, Belgium; 4 Department of Digestive Oncology, Ghent University Hospital, 9000 Gent, Belgium

**Keywords:** image-guided thermal ablation, liver metastases, ablation margin assessment, cone-beam CT hepatic arteriography (CBCT-HA), PET–CT fusion imaging

## Abstract

**Background/Objectives:** Image-guided percutaneous thermal ablation is an established local treatment for selected patients with liver metastases, provided that accurate tumor targeting and adequate ablation margins can be achieved. However, lesion detection, target delineation, and intraprocedural margin verification remain challenging in post-chemotherapy or previously treated lesions that may become morphologically inconspicuous or radiologically occult. Catheter-assisted C-arm (cone-beam) CT hepatic arteriography (CBCT-HA) improves intraprocedural visualization of tumor vascularity and supports streamlined workflows within the angiography suite, yet it may underestimate tumor extent in lesions with limited or absent angiographic conspicuity. This pictorial essay illustrates the feasibility and added value of integrating preprocedural PET/CT with intraprocedural CBCT-HA for liver tumor ablation. **Methods:** Representative clinical cases of percutaneous liver tumor ablation guided by PET–CBCT-HA fusion are presented. Preprocedural PET/CT datasets were rigidly registered and fused with intraprocedural CBCT-HA to support tumor detection, target delineation, ablation planning, and real-time intraprocedural margin assessment. The complementary roles of metabolic and angiographic imaging were evaluated qualitatively across different clinical scenarios. **Results:** PET–CBCT-HA fusion improved detection and delineation of viable tumor components that were occult or insufficiently defined on CBCT-HA alone, particularly in post-chemotherapy or previously treated lesions. Conversely, CBCT-HA identified angiographically evident lesions not apparent on PET/CT. The combined approach enabled confident target definition, biologically informed ablation planning, and immediate post-ablation verification of metabolic and angiographic coverage, supporting margin-oriented intraprocedural decision-making. **Conclusions:** By integrating complementary metabolic and vascular information into a single-session workflow, PET–CBCT-HA fusion represents a multimodal guidance strategy that enhances lesion visualization and intraprocedural margin assessment. This approach may improve local tumor control in complex post-treatment and oligometastatic liver disease.

## Simple Summary

Successful liver tumor ablation depends on clearly identifying all active tumor tissue, accurately placing the ablation probe, and confirming that the treated area fully covers the tumor with an adequate safety margin. This is often challenging after chemotherapy or prior local treatment, when liver metastases may become subtle or invisible on standard imaging. This pictorial overview explains why combining positron emission tomography with catheter-assisted C-arm (cone-beam) computed tomography during ablation may address these limitations. By integrating metabolic information with detailed vascular imaging in a single procedure, this approach can reveal tumors that might otherwise be missed and help verify complete treatment during the intervention. The authors aim to demonstrate how this multimodal strategy supports more precise, margin-focused ablation and may contribute to improved local tumor control in the oligometastatic setting.

## 1. Introduction

Image-guided percutaneous thermal ablation has become an integral component of modern oncologic care for patients with primary and secondary liver tumors [1,2,3,4]. In carefully selected patients with limited liver metastases, randomized controlled trials and prospective cohort studies have demonstrated local tumor control and survival outcomes comparable to surgical resection for lesions up to 3 cm in diameter [5,6].

Parallel to this clinical evolution, liver ablation has transitioned from a purely destructive local intervention toward a form of image-guided precision therapy. Advances in intraprocedural imaging, multimodal image integration, navigation platforms, and ablation monitoring have substantially influenced procedural planning and execution [7,8,9,10,11,12]. Accurate lesion detection, precise applicator placement, and reliable verification of circumferential safety margins are now recognized as key determinants of durable local tumor control.

Among these factors, achieving an adequate peri-ablational safety margin is particularly critical [13,14,15]. The ablation margin, defined as the distance between the original tumor boundary and the outer edge of the ablation zone, has been shown to strongly influence local tumor progression. For small colorectal liver metastases, circumferential margins of at least 5 mm are associated with local recurrence rates below 10% at two years [5,16]. As a result, contemporary ablation workflows increasingly emphasize systematic margin assessment using image registration, fusion, and segmentation tools [17]. Within this framework, intraprocedural margin evaluation may not only reduce the need for retreatment but has also been associated with improved prediction of local tumor progression compared with delayed post-ablation imaging, highlighting the clinical value of real-time treatment verification [18,19].

Selective catheter-assisted intra-arterial contrast injection combined with CT imaging, referred to as CT hepatic arteriography (CTHA), has emerged as a valuable adjunct during liver tumor ablation [20,21]. Compared with conventional CT guidance using intravenous contrast, CTHA provides superior lesion-to-parenchyma contrast, improved delineation of tumor vascularity, and reduced contrast load. However, in centers without a hybrid angiography–CT configuration, conventional CTHA requires patient transfer between angiography and CT imaging suites, introducing workflow inefficiencies and procedural risks. These limitations have been largely mitigated by catheter-assisted C-arm (cone-beam) CT hepatic arteriography (CBCT-HA)-guided ablation, also known as the HepACAGA technique [22,23]. This approach allows arterial catheterization, tumor targeting, ablation, and immediate post-treatment assessment to be performed entirely within the angiography suite. Modern C-arm systems further support advanced navigation and non-axial needle trajectories, facilitating treatment of anatomically challenging lesions.

Despite these advances, lesion visualization can remain challenging, particularly in patients after systemic therapy or prior local treatment, where metastases may decrease in size, lose vascularity, or even radiologically “vanish” on conventional imaging [24]. Quantitative, software-based margin assessment has been shown to outperform visual evaluation alone, reinforcing that objective verification of the minimal ablative margin is central to durable local tumor control [14,15]. Functional imaging with PET/CT provides complementary metabolic information on tumor viability and can improve lesion detection, targeting confidence, and intraprocedural assessment of treatment completeness [25,26,27,28,29]. Recent prospective data further support the importance of biologically informed intraprocedural assessment [29]. Integration of metabolic imaging, biopsy, and margin evaluation improves identification of residual viable tumor and its correlation with ablative margins. Together, these developments support a broader shift from morphology-based ablation toward biologically and quantitatively informed treatment strategies. While real-time PET/CT-guided ablation offers the advantage of direct metabolic visualization, its routine clinical implementation remains limited by technical and logistical constraints [28,30].

In this pictorial essay, we present representative clinical scenarios illustrating how the integration of preprocedural PET/CT with real-time CBCT-HA (PET–CBCT-HA fusion) enhances lesion visualization, target delineation, and ablation margin verification compared with either modality alone. These examples highlight the synergistic role of multimodal image guidance in optimizing local tumor control in complex clinical settings.

## 2. Methods

This pictorial review is based on a retrospective analysis of anonymized imaging data acquired during routine clinical care and includes percutaneous image-guided thermal ablation procedures performed for liver metastases. Cases were selected to illustrate clinically relevant scenarios in which PET–CBCT-HA fusion provided complementary information influencing targeting, planning, or intraprocedural decision-making. The following workflow outlines how preprocedural metabolic information was systematically integrated into intraprocedural angiographic guidance and ablation verification.

All procedures were performed under general anesthesia with high-frequency jet ventilation in a hybrid angiography suite (Artis Icono, Siemens Healthineers, Erlangen, Germany), using a catheter-assisted C-arm CT-guided ablation workflow integrated with PET/CT fusion.

Following femoral arterial access, selective catheterization of the hepatic artery was achieved. An intra-arterial contrast-enhanced cone-beam CT acquired from the hepatic artery (CBCT-HA) was subsequently performed during the arterial phase (DynaCT rotation time of 5 seconds, initiated 8 seconds after the start of contrast injection). This acquisition was routinely used to optimize visualization of primary and secondary liver tumors and to delineate hepatic vascular anatomy.

Preprocedural PET/CT datasets were imported into the angiography workstation and fused with intraprocedural CBCT-HA using a rigid registration workflow based on the CT component of the PET/CT acquisition.

Initial alignment was performed by vendor-provided software within the angiography suite, relying on anatomical landmarks including liver contours, major hepatic and portal veins, and the diaphragm–liver interface. Following automated registration, manual fine adjustments were routinely applied by the operator to optimize alignment, with particular emphasis on the region of interest containing the target lesion. In cases where optimal whole-liver alignment could not be achieved, registration was locally optimized around the tumor-bearing liver segment.

The CT volume was subsequently replaced by the PET dataset to enable metabolic overlay. Fusion adequacy was assessed visually by confirming concordance of intrahepatic landmarks and evaluating spatial agreement between FDG uptake on PET and angiographic features on CBCT-HA at the lesion level. Imperfect intrinsic alignment between the PET and CT components of the PET/CT dataset itself was recognized as a potential source of error. When discordance between CBCT-HA enhancement patterns and PET metabolic activity was observed, careful interpretative adjustment was applied, prioritizing spatial correspondence and relative intensity patterns between PET uptake and CBCT-HA enhancement to achieve the most anatomically plausible alignment.

Ablation planning incorporated both PET-positive tumor extent and angiographic tumor blush, with the aim of achieving a circumferential safety margin of at least 5 mm. The segmented tumor volumes were projected onto live fluoroscopy, enabling continuous intraprocedural visualization during needle navigation and placement.

Needle access was typically obtained using a coaxial technique via an introducer needle, allowing core biopsy sampling and subsequent placement of the microwave ablation antenna along the same trajectory. Needle trajectory planning and stereotactic guidance were performed using the integrated myNeedle Companion navigation platform (Icono VE40, Siemens Healthineers, Erlangen, Germany), with the planned path virtually overlaid on live fluoroscopy. Depending on operator preference and lesion accessibility, needle advancement was performed either manually or with robotic assistance (Micromate, Interventional Systems, Kitzbühel, Austria) to facilitate precise advancement along the predefined trajectory.

Tumor ablation was performed using commercially available microwave ablation systems (Emprint, Medtronic, Minneapolis, MN, USA; Solero, AngioDynamics, Latham, NY, USA; Dophi, SurgNova, Taoyuan City, Taiwan). Immediately after ablation, repeat CBCT-HA with PET fusion was performed to verify complete coverage of all metabolic and angiographic tumor components. Additional ablation was performed when a residual viable tumor was suspected based on metabolic or angiographic findings.

## 3. Results

Figure 1, Figure 2, Figure 3, Figure 4, Figure 5 and Figure 6 illustrate a range of clinically relevant cases demonstrating how the combined use of PET and CBCT-HA provides complementary and actionable information for image-guided ablation. Across all cases, PET–CBCT-HA fusion enhanced lesion detection and characterization. In the first four cases (Figure 1, Figure 2, Figure 3 and Figure 4) involving colorectal liver metastasis, PET/CT was pivotal in identifying viable tumor components that were occult or insufficiently defined on CBCT-HA alone, including post-chemotherapy vanishing lesions, focal local recurrence adjacent to prior ablation zones, and lesions with discordant metabolic and angiographic boundaries.

In contrast, in Cases 5 and 6, CBCT-HA revealed angiographically evident but metabolically occult subcentimetric or satellite lesions not visualized on PET/CT, allowing comprehensive treatment within a single session.

## 4. Discussion

This pictorial essay illustrates how the integration of preprocedural PET/CT with intraprocedural catheter-assisted CBCT-HA can overcome several well-recognized limitations of image-guided liver tumor ablation and may thereby enhance local tumor control. The primary value of this multimodal approach lies in improved delineation of viable tumor tissue and translation of this information into margin-oriented, intraprocedural decision-making.

### 4.1. Tumor Visualization as a Key Determinant of Local Control

Local tumor progression after thermal ablation most commonly results from incomplete targeting of viable tumor tissue or insufficient circumferential ablation margins. These shortcomings are frequently related to limited intraprocedural visualization rather than technical failure of the ablation itself. CBCT-HA enhances lesion-to-parenchyma contrast and provides high-resolution angiographic information, but its performance depends on vascular conspicuity. In post-chemotherapy or previously treated lesions, tumors may become hypovascular, distorted, or radiologically occult, leading to underestimation of viable tumor extent. PET/CT provides complementary metabolic information, enabling detection of viable tumor independent of contrast enhancement or structural visibility. This is particularly relevant in “vanishing” or partially treated lesions, where residual disease may persist despite radiological regression on conventional CT or MRI. In such cases, PET/CT enables localization of metabolically active tumor tissue even in the absence of a clear morphological correlate. Targeting may be guided by contemporaneous PET/CT or, in selected cases, by reference to pre-treatment PET/CT acquired prior to systemic therapy, allowing localization of lesions that have become morphologically occult. Histopathological confirmation of viable tumor in multiple cases reinforces that radiological disappearance does not equate to complete tumor eradication and supports continued consideration of local treatment strategies [24].

### 4.2. Complementary Detection of Metabolic and Angiographic Disease

Importantly, the value of PET–CBCT-HA fusion is bidirectional. While PET enhances identification of viable tumor in morphologically inconspicuous lesions, CBCT-HA provides detailed angiographic information with high spatial resolution that remains essential for procedural execution [23,31]. Hypervascular tumor components, small satellite nodules, and subtle perfusion abnormalities may be angiographically evident despite limited or absent metabolic tracer uptake, particularly in small-volume disease or tumors with intrinsically low avidity. Conversely, PET may reveal viable tumor beyond angiographically apparent boundaries. The integration of both modalities therefore reduces the risk of systematic underestimation inherent to single-modality imaging and supports a more comprehensive assessment of true tumor extent.

### 4.3. Biologically Informed Margin Assessment

Beyond lesion detection, PET–CBCT-HA fusion may enhance intraprocedural assessment of treatment completeness. Fusion of pre-ablation PET data with post-ablation CBCT-HA allows evaluation of whether the ablation zone fully encompasses all metabolically active tumor components. This extends margin assessment beyond purely morphological criteria and may support real-time procedural adaptation, such as additional ablation when coverage appears insufficient. Given the established association between minimal ablative margin and local tumor control, improved intraprocedural margin confidence represents a plausible mechanism by which this approach could contribute to improved outcomes. However, this hypothesis requires validation in prospective outcome-based studies.

### 4.4. Relevance in the Oligometastatic Setting

In the oligometastatic setting, where durable local control is critical, accurate identification and complete treatment of all disease sites are essential. The presented cases illustrate an ongoing shift from morphology-driven targeting toward biologically informed precision therapy. PET–CBCT-HA fusion facilitates integration of functional tumor characteristics throughout all procedural phases, from planning and targeting to intraprocedural verification, within a single intervention. The intraprocedural flexibility, including incorporation of newly identified satellite lesions into the same session, may reduce early local recurrence and potentially delay the need for additional systemic therapy.

### 4.5. Technical Considerations, Limitations and Future Directions

Ablation outcomes in hepatic tumors are fundamentally determined by the ability to localize all viable disease and ensure that the treated volume extends beyond the tumor with an adequate safety margin. Contemporary series on percutaneous thermal ablation of small liver metastases already report low local recurrence rates when treatment is guided by morphological imaging and structured margin assessment. Further improvement may therefore depend on incorporation of functional and biological tumor information into the ablation strategy.

Within this context, PET-CBCT-HA fusion-guided ablation should be interpreted alongside alternative approaches that integrate metabolic information directly into the intraprocedural setting. Real-time PET/CT-guided ablation offers the advantage of direct visualization of metabolically active tumor during the intervention, thereby avoiding reliance on image registration and improving robustness in the presence of respiratory motion, patient repositioning, and liver deformation [28,29,30]. However, PET-based guidance remains limited by spatial resolution, motion artifacts, and relatively limited anatomical detail, necessitating integration with contrast-enhanced CT for accurate localization and assessment of the ablation zone.

PET–CBCT-HA fusion represents a complementary strategy that combines metabolic information with high-resolution angiographic imaging and advanced navigation within the angiography suite. CBCT-HA may improve detection of hypervascular tumor components and small satellite lesions, while PET provides insight into tumor viability beyond angiographic conspicuity. However, fusion-based workflows remain dependent on registration accuracy and do not allow real-time reassessment of metabolic activity after ablation. Interpretation therefore requires awareness of modality-specific limitations, including PET spatial resolution, motion-related artifacts, and the risk of image misregistration. In addition, PET-derived tumor delineation is influenced by segmentation methodology and may not precisely reflect true tumor boundaries [32]. In cases of persistent uncertainty regarding tumor extent or treatment completeness, intraprocedural biopsy may provide complementary information. An integrated approach combining imaging-based, metabolic, and tissue-based confirmation of treatment adequacy, as explored in the OBTABLATE trial, may contribute to striving toward zero local recurrence [29]. Analogous to surgical margin assessment, tissue confirmation may support clinical decision-making, particularly in equivocal lesions or when adequate 5 mm margins cannot be safely achieved.

Given the illustrative and retrospective nature of the present case series, the added value of the PET-CBCT-HA fusion-guided approach should be interpreted as hypothesis-generating rather than definitive. Its clinical benefit is likely most pronounced in complex scenarios with inconspicuous or discordant lesions, where conventional imaging alone is insufficient for confident targeting and margin assessment.

Future validation should therefore focus on prospective, outcome-based studies comparing PET/CT-derived margin assessment with angiographic and morphology-based evaluation. In addition, direct comparison with PET/CT-guided ablation strategies incorporating intraprocedural metabolic feedback may further clarify the relative clinical value of each approach. In its current form, PET–CBCT-HA fusion should be considered a complementary decision-support tool that augments, rather than replaces, real-time anatomical, angiographic and metabolic imaging.

## 5. Conclusions

In summary, PET–CBCT-HA fusion supports a transition from morphology-based ablation toward biologically informed, precision-guided therapy. By improving visualization of viable tumor in complex post-treatment settings, revealing complementary angiographic disease, and enabling real-time margin assessment with adaptive intraprocedural optimization, this multimodal approach provides a plausible mechanistic rationale for improved local tumor control. Consequently, it may contribute to further reductions in local recurrence and support efforts to achieve very low local failure rates in carefully selected patients. Future studies should focus on prospective validation and technical refinement, particularly with respect to image registration accuracy and standardized margin analysis, to better define the role of PET–CBCT-HA fusion in routine metastasis-directed liver ablation.

## Figures and Tables

**Figure 1:**
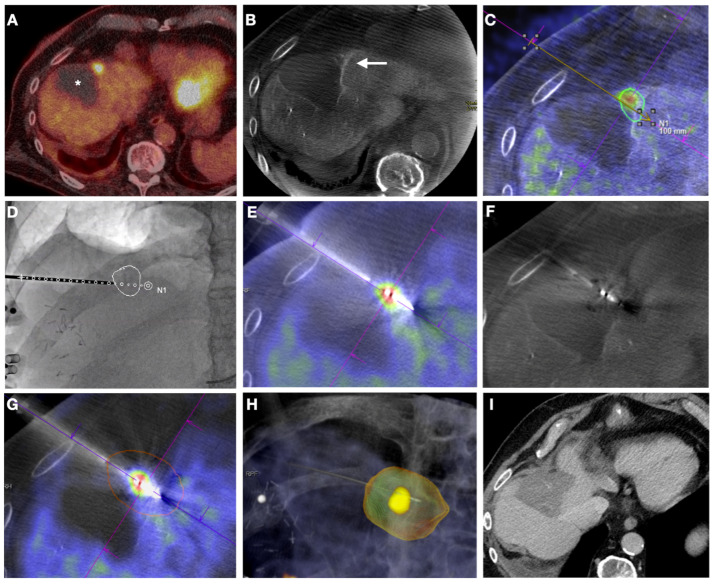
PET–CBCT-HA fusion-guided re-ablation of a focal local recurrence adjacent to a prior liver ablation zone, demonstrating the complementary value of metabolic–angiographic image fusion for accurate target delineation and ablation margin verification. (**A**) FDG-avid focal lesion medial to the prior ablation zone (white asterisks) on PET/CT at 6-month follow-up, consistent with localized recurrence after microwave ablation of a 55 mm liver metastasis. (**B**) Corresponding CBCT-HA image showing subtle rim enhancement (white arrow) with indistinct margins due to post-ablation perfusion alterations, limiting reliable morphological delineation. (**C**) PET–CBCT-HA fusion-based segmentation of the metabolically active tumor used for intraprocedural navigation. (**D**) Stereotactic placement of the microwave ablation antenna along the planned trajectory under fused imaging guidance. (**E**) PET–CBCT fusion confirming accurate antenna positioning within the FDG-avid target lesion. (**F**) Immediate post-ablation imaging demonstrating a confluent necrotic ablation zone. (**G**) Fusion of the segmented post-ablation necrotic zone with pre-ablation PET avidity, confirming complete coverage with a minimal circumferential ablation margin exceeding 5 mm. (**H**) 3D rendering of the segmented tumor and ablation zone illustrating spatial coverage. (**I**) Contrast-enhanced CT at 6-month follow-up demonstrating complete local tumor control.

**Figure 2:**
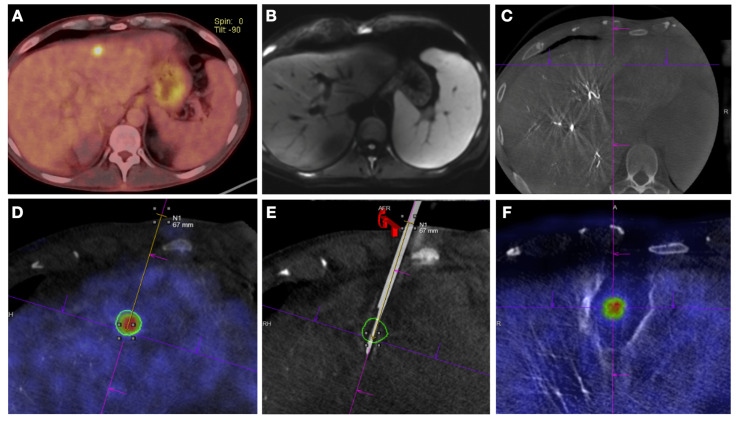
PET–CBCT-HA fusion-guided ablation of a vanishing colorectal liver metastasis after systemic therapy, demonstrating the value of metabolic guidance in the absence of morphological correlates. (**A**) FDG-avid colorectal liver metastasis in segment II on baseline PET/CT. (**B**) Post-chemotherapy diffusion-weighted MRI showing radiologic disappearance of the lesion. (**C**) CBCT-HA failing to demonstrate a discernible lesion at the corresponding location. (**D**) PET–CBCT-HA fusion-based segmentation of the metabolically active target used for intraprocedural navigation. (**E**) CBCT fusion confirmed accurate biopsy needle positioning within the target (confirming residual metastasis), and the MWA antenna was placed coaxially through the same introducer. (**F**) Post-ablation PET–CBCT–HA fusion confirming A0 ablation with a circumferential safety margin exceeding 5 mm.

**Figure 3:**
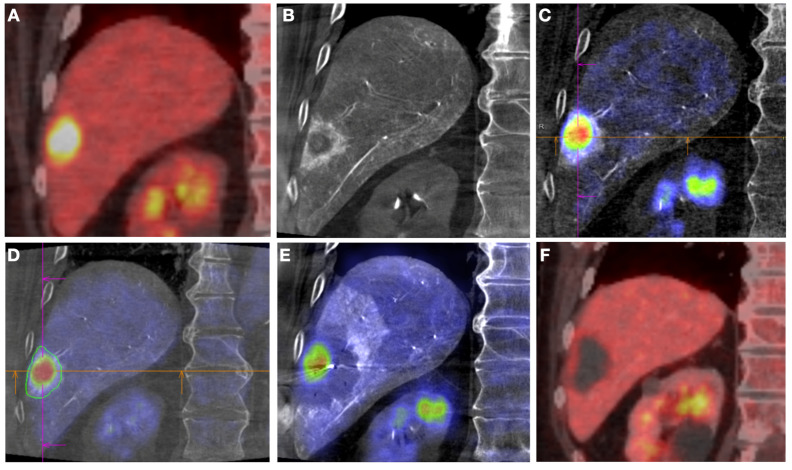
Metabolic–angiographic image fusion for defining true tumor extent in a hypervascular colorectal liver metastasis with discordant angiographic and metabolic boundaries. (**A**) FDG-avid subpleural colorectal liver metastasis in segment VI on PET/CT. (**B**) CBCT-HA demonstrating a prominent hypervascular rim with ill-defined lesion boundaries. (**C**) PET–CBCT-HA fusion showing only partial overlap between metabolic activity and angiographic enhancement. (**D**) Fusion-based lesion segmentation refining the target volume. (**E**) Post-ablation imaging demonstrating complete coverage of the FDG-avid component with a circumferential margin exceeding 10 mm. (**F**) PET/CT at 1-year follow-up confirming complete metabolic response.

**Figure 4:**
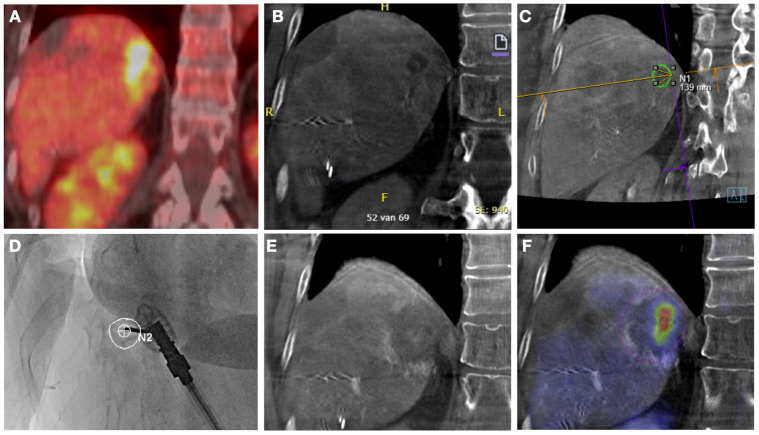
PET-guided expansion of the ablation volume in a hypovascular colorectal liver metastasis with PET-positive extension beyond angiographically visible boundaries. (**A**) Elongated FDG-avid colorectal liver metastasis in segment VII on PET/CT. (**B**) CBCT-HA showing a smaller hypovascular lesion, which may either represent an underestimation of the true tumor extent or reflect apparent FDG uptake beyond the true tumor margins due to respiratory motion-related artifacts. (**C**) Planning of the optimal needle trajectory for navigation toward the CBCT-HA-segmented target. (**D**) Stereotactic placement of the microwave ablation antenna using needle guidance, with the planned trajectory virtually superimposed on the fluoroscopic image. (**E**) Immediate post-ablation CBCT-HA demonstrating the large ablation zone. (**F**) PET–CBCT-HA fusion confirming complete coverage of the entire FDG-avid tumor component with a minimal safety margin greater than 5 mm.

**Figure 5:**
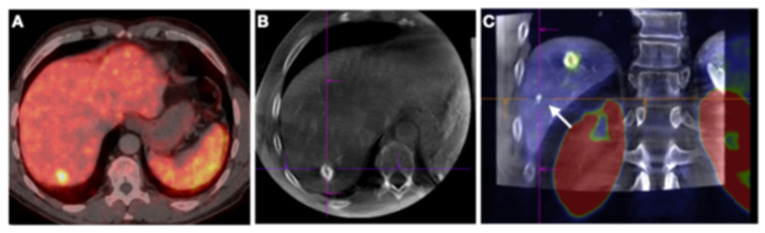
CBCT-HA detection of angiographically evident but metabolically occult hepatic metastasis in oligometastatic prostate cancer, enabling comprehensive single-session ablation. (**A**) PSMA-avid prostate cancer liver metastasis in segment VII on PET/CT. (**B**) Corresponding hypervascular lesion on CBCT-HA. (**C**) PET–CBCT-HA fusion revealing an additional subcentimetric hypervascular metastasis (white arrow) without PSMA uptake, which was incorporated into the same ablation session. Post-procedurally, PSA levels declined to undetectable values for the first time in several years.

**Figure 6:**
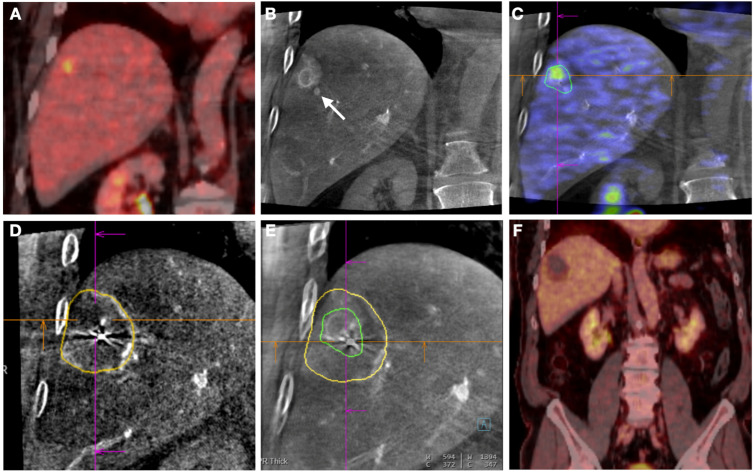
Identification and treatment of a clinically relevant satellite lesion in oligometastatic melanoma using combined PET–CBCT-HA guidance. (**A**) FDG-avid melanoma liver metastasis in segment VII on PET/CT. (**B**) CBCT-HA clearly delineating the target lesion and revealing an additional millimetric satellite focus (white arrow) not visible on PET/CT. (**C**) PET–CBCT-HA fusion-based segmentation including both lesions within a single target volume. (**D**) Segmentation of the post-ablation necrotic zone. (**E**) Pre- and post-CBCT-HA fusion confirming circumferential ablation margins exceeding 5 mm around both lesions. (**F**) PET/CT at 3-month follow-up demonstrating complete metabolic remission.

## Data Availability

Data supporting the findings of this study are available from the corresponding author upon reasonable request.
